# Checkpoint inhibitor immunotherapy induced inflammatory arthritis secondary to Nivolumab and Ipilimumab: a pediatric first

**DOI:** 10.1186/s12969-024-00983-3

**Published:** 2024-04-29

**Authors:** John A. Storwick, Herman Tam, Daniel G. Rosenbaum, Kristin Houghton

**Affiliations:** Division of Pediatric Rheumatology, Department of Radiology, British Columbia Children’s Hospital, The University of British Columbia, Vancouver, Canada

**Keywords:** Immune checkpoint inhibitors, irAE, Pediatric, Inflammatory arthritis, Nivolumab, Ipilimumab

## Abstract

**Background:**

Immune checkpoint inhibitors (ICIs) have expanded the arsenal of cancer therapeutics over the last decade but are associated with a spectrum of immune-related adverse events (irAEs), including inflammatory arthritis. While these complications are increasingly recognized in the adult population, no cases of inflammatory arthritis irAEs have been reported in the pediatric literature.

**Case Presentation:**

A 14-year-old female with metastatic epithelioid mesothelioma was referred to the pediatric rheumatology clinic after developing progressive inflammatory joint pain in her bilateral shoulders, hips, and small joints of hands following the second cycle of Nivolumab and Ipilimumab. Initial examinations showed bilateral shoulder joint line tenderness, positive FABERs test bilaterally, tenderness over bilateral greater trochanters, and bilateral second PIP effusions. Her serological profile was notable for positive HLA-B27, positive anti-CCP, negative Rheumatoid Factor, and negative ANA. PET-CT scan performed for disease response following immunotherapy showed symmetric increased metabolic activity primarily involving the supraspinatus, gluteus medius and minimus, and semimembranosus tendon insertions. Her presentation was consistent with a grade 1 irAE that worsened to a grade 2 irAE despite NSAID therapy, prompting a short course of oral prednisolone. She achieved clinical remission of her mesothelioma following six cycles of Nivolumab and Ipilimumab and her inflammatory arthritis was controlled on Celebrex monotherapy.

**Conclusions:**

To our knowledge, this is the first pediatric case of ICI-induced inflammatory arthritis and enthesitis. This case highlights the importance of increasing awareness of diagnosis and management of irAEs in children.

## Background

Over the last decade, immune checkpoint inhibitor (ICI) therapy has expanded the arsenal of cancer therapeutics in adults. Nivolumab is a human IgG4 monoclonal antibody against PD-1 that binds to PD-1 receptors found on T lymphocytes, thus blocking inhibition of T cell proliferation and cytokine production, resulting in amplified antitumor immunity. Ipilimumab is a humanized IgG1 monoclonal antibody that inhibits CTLA4, resulting in prolonged T-cell activation, amplification of T-cell-mediated immunity, and improved antitumor immune response [1]. Both Nivolumab and Ipilimumab are approved for treating mesothelioma in adult patients and have been associated with developing inflammatory arthritis, a category of complication designated an immune-related Adverse Event (irAE) [2]. 

Although beneficial in improving oncologic outcomes, ICIs are associated with a spectrum of irAEs. ICIs have different toxicities than conventional chemotherapy, and most irAEs result from increased immune reactivity. As a result, irAEs can affect a wide range of organ systems, including rheumatic-irAEs (Rh-irAE) such as inflammatory arthritis, myositis, polymyalgia-like syndrome, sicca syndrome, systemic lupus erythematosus, sarcoidosis, and vasculitis. irAEs are described using the Common Terminology Criteria for Adverse Events (CTCAE). They are graded based on severity, with Grades 1 and 2 being considered mild, grades 3 and 4 as severe, and grade 5 indicating that the patient died due to the adverse event (Table 1) [3, 4]. 

We present the case of a 14-year-old girl with progressive inflammatory arthritis secondary to Nivolumab and Ipilimumab therapy. A literature review through PubMed was completed using the search terms “irAE” “checkpoint inhibitor” “Pediatric”, “Ipilimumab”, “Nivolumab”, and “inflammatory arthritis”. It showed no published cases of ICI-induced inflammatory arthritis in the pediatric population.

We therefore believe this to be the first published case of inflammatory arthritis and enthesitis caused by ICI therapy in the literature to date.

## Case Presentation

A now 14-year-old female of Caucasian descent was diagnosed with metastatic epithelioid mesothelioma at age 13 following initial presentation with deep vein thrombosis and constitutional symptoms. Abdominal ultrasound and MRI imaging showed pelvic peritoneal nodularity, and an initial PET scan showed low-grade increased FDG avidity in the area. Biopsy of the nodules was positive for epithelioid mesothelioma. Subsequent imaging raised concerns for chest deposits, and a biopsy confirmed thoracic involvement. Initial treatment of 6 cycles of cisplatin/pemetrexed showed a partial favorable therapeutic response with decreased metastatic burden but residual thoracic lymphadenopathy, and pleural and peritoneal disease. During her last dose of cisplatin, she developed a severe anaphylactic reaction. Given remaining PET avidity in both her abdomen and chest, additional systemic therapy was initiated with ‘6-weekly’ cycles of Nivolumab on day 1 and Ipilimumab on days 1 and 22 of each cycle.

**Fig. 1:**
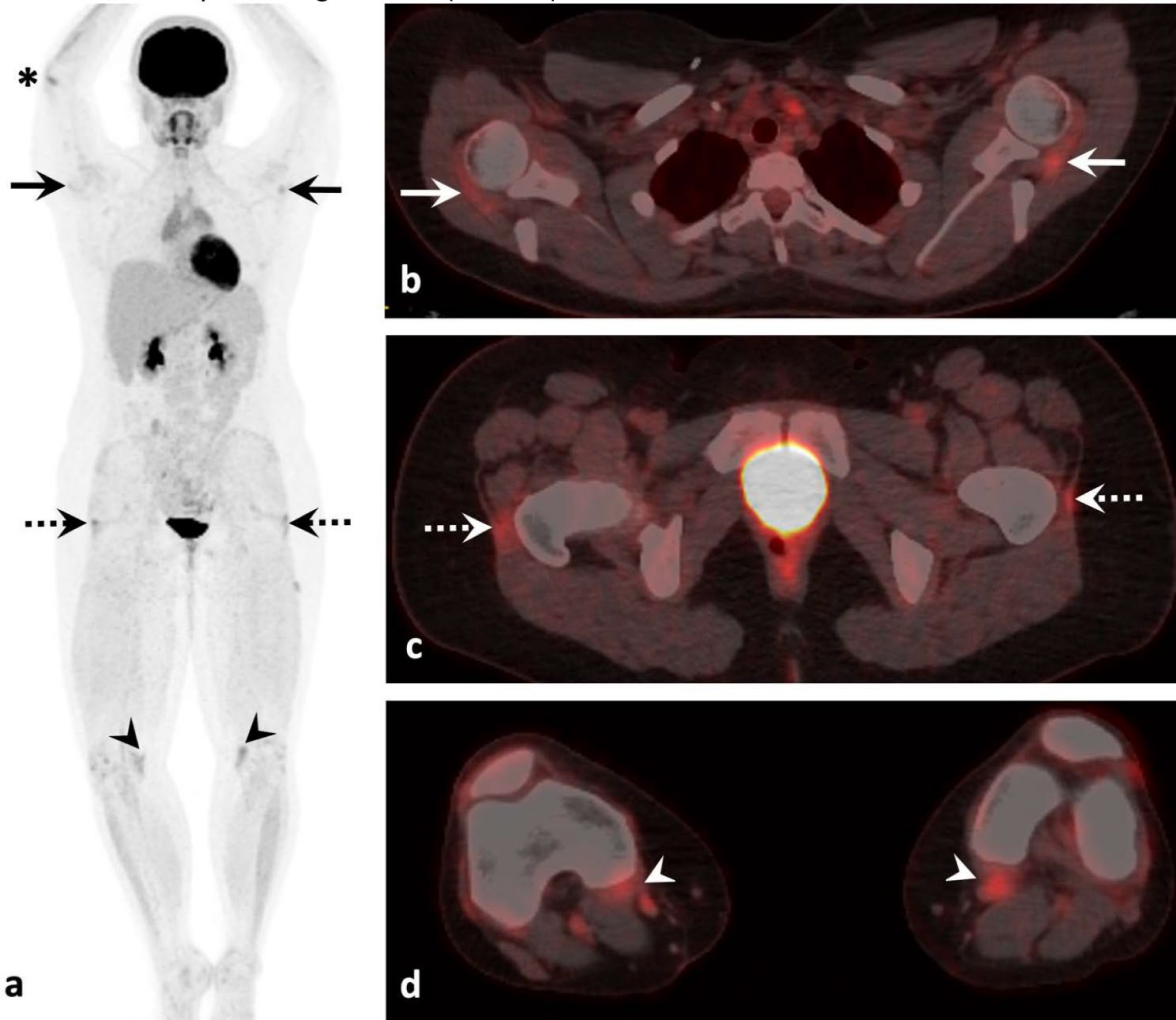
Whole body PET-CT following initiation of immunotherapy. Maximum intensity projection (**a**) and axial fused images (**b-d**) demonstrates symmetric increased metabolic activity at the bilateral supraspinatus (*solid arrows*), gluteus medius/minimus (*dashed arrows*), and semimembranosus (*arrowheads*) tendon insertions. There is also focally increased metabolic activity at the right elbow (*asterisk*)

Following her second cycle of Nivolumab and Ipilimumab, she developed joint pain and stiffness, mainly located in her right elbow, bilateral shoulders, second metacarpal-phalangeal joints, proximal interphalangeal joints, knees, and hips. Her hip pain was associated with 15–20 min of morning stiffness without limitation of her range of motion. She had no functional impairment at the time and could continue with her regular activities. Following her 3rd cycle of Nivolumab and Ipilimumab, a repeat PET-CT scan showed a complete response of her mesothelioma. However, it also showed new symmetric increased metabolic activity at multiple entheses in the shoulders, hips, knees, and right elbow, most pronounced involving the supraspinatus, gluteus medius and minimus, and semimembranosus tendons (Fig. 1).

She was subsequently referred to our pediatric rheumatology clinic for assessment of potential irAE secondary to Nivolumab and Ipilimumab. Her initial examinations in the clinic showed bilateral shoulder joint line tenderness, positive FABERs test bilaterally, tenderness over bilateral greater trochanters, and effusions of her bilateral second PIPs of her hands. Her serological profile was notable for positive HLA-B27, positive anti-CCP, negative Rheumatoid Factor, and negative ANA. Her family history was negative for rheumatic conditions, aside from osteoarthritis in her mother. She had no prior joint symptoms to suggest a pre-existing rheumatic condition.

At her initial consultation, she was diagnosed with a grade 1 Rh-irAE with mild pain and clinical arthritis [4]. She was initially treated with Naproxen 10 mg/kg/dose twice daily with good improvement; however, with each subsequent cycle of ICI therapy, her inflammatory arthritis worsened despite escalating naproxen doses up to 12 mg/kg/dose twice daily.

Following her fifth cycle of ICI therapy, her symptoms worsened to a grade 2 Rh-irAE with worsening of her pain and new limitations of her daily activities. She was subsequently started on oral prednisolone 0.5 mg/kg/day, and her Naproxen was changed to Celecoxib due to GI intolerance. She had significant improvement in her pain and function after starting systemic steroids. Her steroids were tapered over a 3-month period without flaring of her inflammatory arthritis. At her last follow-up, she had achieved clinical remission of her mesothelioma following six cycles of Nivolumab and Ipilimumab and had control of her inflammatory arthritis on Celecoxib monotherapy.

## Discussion and Conclusion

Among adults, inflammatory arthritis is a known side effect of immune checkpoint inhibitor therapy and is demonstrated in several case series and retrospective cohort studies [5–9]. Our case represents the first report to our knowledge of inflammatory arthritis associated with Nivolumab and Ipilimumab therapy in a child.

Given the lack of pediatric cases, in discussion with our oncology colleagues, we based our management choice on the adult literature. A suggested management algorithm of Rh-irAEs in adults is directed by the CTCAE gradings, with grade 1 being treated with analgesia and non-steroidal anti-inflammatory drugs; grade 2 being treated with a short course of oral steroids without discontinuation of ICI therapy; and grade 3 and above being treated with high-dose oral or intravenous steroids and discontinuation of ICI. The goal of treatment is to minimize the impact of Rh-irAEs while allowing for ongoing oncologic immunotherapy. Given their slow onset of action, early initiation of disease-modifying antirheumatic drugs (DMARD) therapy is considered beneficial if patients cannot wean off steroids without recurrence of their irAE symptoms [10]. 

Given the expanding field of immune checkpoint inhibitors in pediatric oncology, we predict an increase in irAEs will be seen by pediatric rheumatologists in the coming years. This case highlights the importance of increasing awareness of Rh-irAEs in children. Although basing management of Rh-irAEs on adult algorithms achieved an excellent clinical outcome in our case, gathering further data will help determine the optimal management strategy of Rh-irAEs in children and if they differ from adults. Pediatric rheumatologists’ involvement in the multidisciplinary management of Rh-irAEs will be crucial in the coming years.

**Table 1:** CTCAE Grading System [3, 4]

Grade	Severity of Adverse Event
Grade 1	Mild; asymptomatic or mild symptoms; clinical or diagnostic observations only; intervention not indicated
Grade 2	Moderate; minimal, local, or noninvasive intervention indicated; limiting age-appropriate instrumental ADL*
Grade 3	Severe or medically significant but not immediately life-threatening; hospitalization or prolongation of hospitalization indicated; disabling; limiting self-care ADL**
Grade 4	Life-threatening consequences: urgent intervention indicated
Grade 5	Death related to adverse events

↵* Instrumental ADL (activities of daily living) refers to preparing meals, shopping, managing money, etc.

↵** Self-care ADL refers to bathing, dressing, feeding, toileting, etc.

## Data Availability

Not applicable.

## Abbreviations

ICI: immune Checkpoint Inhibitors
irAE: immune-related adverse events
ANA: antinuclear antibody
CTCAE: Common Terminology Criteria for Adverse Events
DMARD: disease-modifying antirheumatic drugs
Anti-CCP: anti cyclic citrullinated peptide
